# Real-World Safety and Efficacy of Venetoclax in Chronic Lymphocytic Leukemia: A Single-Center Comparative Analysis With Randomized Clinical Trials

**DOI:** 10.1155/ah/3910332

**Published:** 2025-08-19

**Authors:** Sophie Thau, Christian Bjørn Poulsen, Morten Kranker Larsen, Lars Møller Pedersen

**Affiliations:** ^1^Department of Hematology, Zealand University Hospital, Roskilde, Denmark; ^2^Department of Clinical Medicine, University of Copenhagen, Copenhagen, Denmark

## Abstract

Treating chronic lymphocytic leukemia (CLL) with the BCL-2 inhibitor venetoclax has shown favorable results in randomized clinical trials (RCTs). Regulatory authorities have recognized the need for also investigating the efficacy and safety of new antineoplastic therapies in real-world (RW) studies with patients often characterized by higher age and comorbidities than patients treated in RCTs. We present a RW single-center study of 112 patients with CLL or small lymphocytic lymphoma (SLL) treated with venetoclax at Zealand University Hospital. A total of 74 patients were treated according to the standard clinical practice and 38 were included in RCTs. No significant differences in efficacy profiles, or safety measures were observed between the two cohorts. Both groups presented overall acceptable tolerability and safety profiles to venetoclax. Moreover, our results suggest that tumor lysis syndrome (TLS) was not a clinical challenge in RW patients even when 6- and 12 h blood samples for TLS were omitted. RW CLL/SLL patients treated outside a clinical trial also had comparable safety and efficacy profiles as reported in the MURANO, CLL13, and CLL14 trials. In conclusion, patients with CLL treated with venetoclax in a RW clinical setting exhibit similar efficacy and safety outcomes to those observed in RCTs.

## 1. Introduction

Over the past decade, the therapeutic landscape has shifted from chemoimmunotherapy to targeted therapy in patients with chronic lymphocytic leukemia/small lymphocytic lymphoma (CLL/SLL). The BCL-2 inhibitor venetoclax in combination with an anti-CD20 monoclonal antibody has been tested in three pivotal randomized clinical trials (RCTs) with CLL/SLL patients, the MURANO study and the CLL13 and CLL14 trials [1–3]. In the MURANO trial, the combination of rituximab and venetoclax was compared to chemoimmunotherapy in relapsed or refractory (R/R) CLL patients. [1]. The venetoclax-based therapy had significantly longer progression free survival (PFS) and overall survival (OS), and the benefit persisted in a 7-year update [4, 5]. In the CLL14 trial, venetoclax and obinutuzumab was compared to chlorambucil and obinutuzumab in first-line treatment of patients with coexisting conditions (“unfit”) [2]. The venetoclax combination resulted in a significantly longer PFS which was sustained in the 6-year follow-up [6]. The CLL13 trial included “fit” CLL/SLL patients, and first-line standard chemoimmunotherapy was compared to venetoclax in combination with rituximab, obinutuzumab, or obinutuzumab plus ibrutinib. Venetoclax–obinutuzumab combinations were superior to chemoimmunotherapy in relation to PFS [3]. In these key RCTs, adverse events (AEs) Grades 3-4 occurred in 69%–82% of patients, with the most frequent events being neutropenia, anemia, and infections [1–3]. A potential serious side effect is tumor lysis syndrome (TLS), which has been reported as a potential life-threatening complication to venetoclax treatment [7]. Therefore, a ramp-up dosing schedule has been developed and is recommended with risk assessment strategies to identify patients in high risk of developing TLS [8].

Real-world (RW) studies include a wider range of patients, reflecting more diverse clinical settings and patient populations. The value of RW data has been recognized by regulatory authorities such as the US Food and Drug Administration (FDA) and the European Medicines Agency (EMA) [9, 10]. While RCTs minimize bias and confounding through randomization and strict inclusion and exclusion criteria, this internal validity often compromises generalizability since RCT populations may differ from patients encountered in everyday practice including elderly patients and those with multiple comorbidities. Thus, toxicity and outcomes have been differently reported comparing data from RW studies and clinical trials as demonstrated in CLL patients treated with kinase inhibitors [11]. A RW study in patients with acute myeloid leukemia treated with venetoclax reported a significantly lower response rate in the RW context compared to patients treated in the VIALE—A trial emphasizing the importance of utilizing RW studies to support the findings of clinical trials [12]. RW data evaluating effectiveness and safety of venetoclax-based regimens in CLL are limited. As venetoclax has emerged as a pivotal treatment for CLL, the demand for RW evidence to validate its efficacy and safety has grown significantly. In this study, we present findings from a single-center experience with CLL/SLL patients treated with venetoclax-based regimens in standard clinical practice and RCT settings.

## 2. Methods

### 2.1. Patients

Patients diagnosed with CLL/SLL and treated with venetoclax in the period April 2017 to January 2023 at Zealand University Hospital were identified in the electronic patient platform (Epic). Patients ≥ 18 years diagnosed with CLL/SLL and treated with a venetoclax-based regimen were included. Patients with verified Richter transformation before initiation of venetoclax were excluded. The study was approved by the regional Data Protection Authority (ID: EMN-2022-11065). Patient consent was provided by patients alive at follow-up.

### 2.2. Data Collection

We conducted a detailed review of medical records. Clinical information was collected including demographics, results of blood analyses, clinical and genetic prognostic factors, imaging and pathology reports, CLL treatments, comorbidity burden, venetoclax dosage, treatment toxicity, TLS events and management, treatment responses, and survival. Diagnosis, staging, and response assessment were conducted according to the iwCLL guidelines [13]. Comorbidity was rated using the Cumulative Illness Rating Scale (CIRS) score [14]. TLS was graded 0–5 according to Cairo–Bishop criteria revised by Howard et al. [15]. TLS was monitored from the time of initiation of venetoclax until the venetoclax ramp-up dosing schedule had been completed. TLS biochemistry was obtained according to the Danish CLL Society recommendations for venetoclax ramp-up, which only requires predose and 24-h post-dose TLS monitoring in intermediate and high-risk patients. AEs were graded 0–5 according to NCI Common Toxicity Terminology Criteria for AEs v5.0 (NCI CTCAE). Side effects were defined according to treatment-emergent adverse events (TEAE) not present prior to treatment with venetoclax, or an already present event that worsened in either intensity or frequency following treatment. The treatment-emergent period was defined from the time of the first dose of venetoclax through the date of last dose plus 30 days or the day before initiation of a new antineoplastic treatment, whichever occurred first.

### 2.3. Statistics

Response assessment was recorded as the best response, reflecting the most favorable outcome observed up to the point of follow-up. Complete remission (CR) included CR with incomplete hematologic recovery. Overall response rate (ORR) was calculated as the proportion of patients with CR or partial response (PR). Other outcome measurements were PFS and OS calculated from the initiation of venetoclax. Last follow-up was censored at the time of the end of study. Further endpoints were venetoclax discontinuation, dose reduction, and achieved venetoclax maximum dose (400 mg). Treatment discontinuation was defined as ending therapy for all reasons other than planned completion of therapy. Endpoints of TLS-associated toxicity were biochemical TLS, clinical TLS, TLS-associated death, and frequency of hemodialysis. Due to a modest number of patients in subgroups, comparisons are descriptively summarized. Categorical variables are presented as numbers (percentages) and continuous variables as median (ranges).

## 3. Results

### 3.1. Patient Characteristics

We identified 821 patients with CLL/SLL, of whom 112 had received venetoclax-based treatment at some point. The number of patients treated with venetoclax in RCTs (RCT cohort) was 38 (34%), while 74 (66%) received treatment according to the standard clinical practice (RW cohort). Clinical characteristics of the total study cohort and according to whether patients were treated in a nonprotocol or RCT context are summarized in Table 1. The frequencies of treatment-naïve CLL/SLL patients in the RCT and RW cohorts were 45% and 31%, respectively. We found no obvious differences in clinical characteristics between the groups, although patients treated outside protocol tended to be older and had more frequent pulmonary disease. The RCT group had a trend toward fewer patients with Binet Stage C and *TP53* mutation, while other molecular characteristics were similarly distributed.

### 3.2. Treatment Characteristics

Characteristics of the venetoclax treatment are shown in Table 2. Combinations with a BTK-inhibitor including ibrutinib and acalabrutinib were exclusively used in patients treated in a clinical trial, while venetoclax monotherapy was only applied in patients treated outside protocol. Venetoclax was preplanned to be administered for 12 and 24 months in 48% and 52%, respectively. Patients treated in the RW cohort had a slightly higher frequency of R/R disease. All patients followed a dose ramp-up schedule of 5 weeks. Number of patients achieving maximum daily dose (400 mg) after completion of venetoclax ramp-up was 87% in patients outside of a trial and 90% within a RCT. The duration of exposure to venetoclax and the need for dose reduction or premature discontinuation were also comparable between the groups. More patients in the RCT cohort received supportive growth factors and antibiotic prophylaxis. Other treatment characteristics were similarly distributed between the groups.

### 3.3. Safety

**Figure 1:**
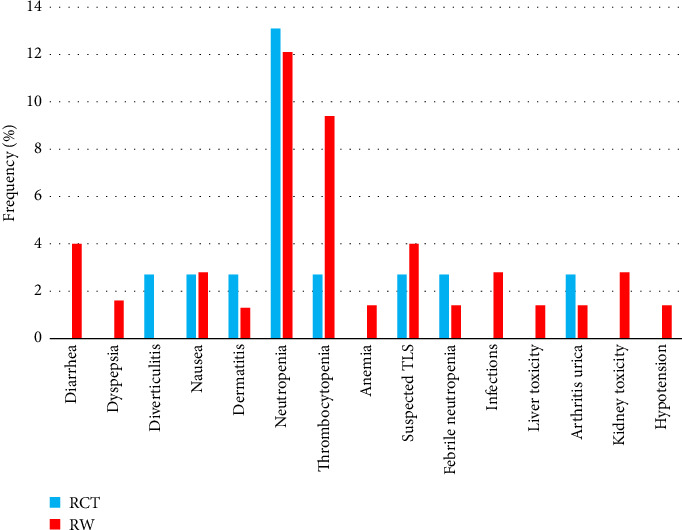
Treatment-emergent adverse events (TEAEs) during ramp-up period (5 weeks) in the study cohort of patients treated with venetoclax in clinical trials (RCT, *n* = 38) and outside clinical trials (RW, *n* = 74).

The frequencies of patients with at least one Grades 3-4 AE were 58% and 63% in the group of patients treated in clinical trials and according to standard practice, respectively. Grades 3-4 toxicities are detailed in Table 3. The frequency of pneumonia Grades 3-4 was higher in patients treated in routine practice. In the subgroup of patients with pneumonia as a Grades 3-4 adverse event, there was a tendency toward a higher proportion of patients with advanced-stage CLL, neutropenia, and unmutated IGHV status. However, due to the limited number of cases, statistical associations could not be reliably assessed. No other differences in the distribution of toxicity were demonstrated. TEAEs were recorded in 30 patients (27%). TEAEs during venetoclax ramp-up in our total study cohort are shown in Figure 1. Neutropenia was the most frequent TEAE (46%). The hospitalization rate during the ramp-up period was 18%. No substantial differences in the distribution of TEAEs and hospitalization rate were observed between the RCT and RW groups.

Blood analyses for TLS assessment were collected before the initiation of venetoclax in 112/112 (100%), at 6 h in 39/112 (35%), at 12 h in 5/112 (4%), and at 24 h in 109/112 (97%) samples. Biochemical TLS Grades 1-2 was observed in only two patients (1.7%), both assessed as having an intermediate risk for TLS before treatment. No patients presented with biochemical TLS Grades 3-4 or clinical TLS. TLS prophylaxis was administered in 111 (99%) patients with two patients receiving rasburicase, and the remaining 109 patients a xanthine oxidase inhibitor (allopurinol) or uricosuric agent (probenecid). No patients underwent debulking strategies prior to venetoclax.

### 3.4. Efficacy

The ORR for the entire cohort was 94%, with 74% achieving a CR. In three patients (4%) who were treated outside of a clinical trial, the response could not be evaluated because venetoclax treatment was prematurely discontinued at a very low dose level (< 50 mg) for less than four weeks. The ORR in the group of patients treated in a clinical trial was comparable to patients treated in the RW cohort (100% vs. 91%). There was a trend toward a higher CR rate in the RCT cohort compared to patients treated in a RW setting (84% vs. 69%). Median (range) time from initiation of venetoclax to follow-up was 18 months (1–36) for the entire study cohort. The OS rate was slightly higher in the RCT group than in the RW group, with 2-year rates of 100% and 89%, respectively. PFS were comparable in the two groups, with 2-year PFS rates of 91% in RCT patients and 87% in RW cohort.

## 4. Discussion

We report RW data on safety and efficacy from a monocenter setting of 112 CLL/SLL patients treated with venetoclax in RCTs or according to RW clinical practice. In general, patients treated in our standard practice cohort and in clinical trials had similar clinical baseline characteristics, safety profiles, and efficacy. The RW data were also comparable to previously reported data in the key multicenter RCTs. However, patients treated in our routine setting tended to be older with a higher frequency of advanced disease stage. In the MURANO trial with R/R patients and the CLL13 trial with “fit” treatment-naïve patients, the median age of the study populations was 64 and 62 years, respectively, compared to a median age of 74 years in our RW routine setting [1]. In the MURANO, CLL13, and CLL14 trials, 25%–43% of the patients had Binet Stage C or Rai Stage III-IV, while 54% of our RW patients were Binet Stage C [1–3]. The R/R patients in our RW setting were also more heavily pretreated than patients in the MURANO trial [1]. In contrast, the frequency of patients in the RW cohort with *TP53* mutations or 17p deletion was similar to patients reported in the RCTs [1–3]. A history of severe organ disease of any kind would be considered an exclusion criterion in most RCTs, and therefore, one could expect that more patients in a real-life setting are characterized by a higher comorbidity burden. Comorbidity was not reported according to the CIRS score in the MURANO trial, while the CLL13 and 14 studies were aimed at patients with low and high comorbidity burden, respectively. However, when comparing our single-institution RW and RCT cohorts, there was a comparable level of comorbidity.

We found similar frequencies of our RW routine, and RCT patients that received the preplanned maximum dose level of venetoclax and treatment duration were evenly distributed. In our cohort, 29% were reduced in the steady-state venetoclax dose and 24% discontinued venetoclax prematurely with no obvious differences between patients in a routine versus clinical trial setting. In the MURANO trial with R/R patients, 19% had venetoclax discontinued prematurely for reasons other than disease progression or death, while the discontinuation rate was only 16% due to toxicity in “unfit” patients participating in the CLL14 trial [1, 2]. Our data suggest that dose intensity and adherence to treatment with venetoclax might be slightly lower in a RW setting.

Neutropenia was the most frequently reported Grade 3-4 AE in the key RCTs, ranging from 57% in R/R patients in the MURANO trial to 42% of “fit” patients in the CLL13 trial receiving first-line treatment with venetoclax and rituximab or obinutuzumab [1–3]. In comparison, 46% experienced Grades 3-4 neutropenia in our study population, including 51% of R/R patients treated outside a RCT. However, we found a higher fraction of Grades 3-4 infections in our RW patients treated outside a clinical trial with a frequency of 49% among R/R patients which in the MURANO study was reported to be only 18% [1]. Pneumonia was the predominant infection in our RW setting, while febrile neutropenia Grades 3-4 was recorded in only 4% of the patients. Use of supportive treatment with granulocyte colony-stimulating factor was equally distributed when comparing data from RCTs with our RW patients, but the incidence of infections was higher in the RW cohort. More patients in the MURANO group received prophylactic antibiotics, and the higher incidence in the RW cohort suggest that the use of prophylactic antibiotics might be reasonable also in a RW setting. A more detailed analysis of risk factors was not feasible in this relatively small cohort of patients. Strategies for infection prevention should be evaluated in the context of RCTs.

TLS data on patients treated with venetoclax outside clinical trials are heterogenous, with two large retrospective analyses reporting a TLS incidence between 5% and 10%, and in a recent study, an incidence of TLS observed even higher at 13% [16–18]. The frequency of Grades 3-4 biochemical TLS was 3.1% in the MURANO trial with only one case of clinical TLS recorded [1]. In the CLL13 trial, the rate of clinical TLS Grades 3-4 was 1.7% [3]. In our RW cohort, we found a very low incidence of TLS with no cases of clinical TLS and only two cases of biochemical TLS Grades 1-2 indicating that TLS can be managed safely in a RW setting with the necessary preventive measures during ramp-up of venetoclax. In our RW patients, routine practice did not include collecting the 6- and 12-h blood samples for TLS monitoring. Our real-life data support that this strategy can be used without compromising safety if 24-h samples are monitored in patients with intermediate or high risk of TLS. Debulking strategies prior to venetoclax initiation have been reported to imply a reduced risk of TLS [19]. In the present study, no debulking strategies were used to prevent severe TLS. According to Danish national recommendations, however, venetoclax should be initiated before the addition of obinutuzumab to reduce the risk of infusion-related reactions (IRR). This approach does not appear to increase the risk of TLS.

Comparison of data on efficacy between our RW cohort and RCTs is challenging due to significant population matching discrepancies [1–3]. Furthermore, MRD-negativity was a mandatory criterion for achieving a CR in the RCTs, and direct comparisons of response rates were not feasible. However, we could demonstrate a tendency toward higher CR rates in our own group of patients treated in a clinical trial compared to our patients treated in a routine setting. In contrast, our data suggest comparable ORR, PFS, and OS in the RW R/R patients compared to patients in the MURANO trial, despite the RW cohort being in a higher age group and with more patients having advanced disease stage [1]. Our study provides RW data reflecting routine clinical practice, offering a complementary perspective to findings from RCTs. Although the small sample size and expected differences in patient characteristics limit direct comparative analyses, our results are consistent with existing evidence that patients treated with venetoclax in RW settings can achieve outcomes comparable to those reported in RCTs.

Previous studies of venetoclax treatment in a RW setting have primarily reported data on R/R CLL patients with prognostically unfavorable characteristics who have received more treatments before venetoclax. However, a prospective observational multicenter study investigated safety and efficacy of the combination of venetoclax and obinutuzumab in first-line therapy, showing data comparable to the CLL14 trial [20]. The ORR was 91% with 34% experiencing Grades 3-4 AEs. In a recent study of R/R patients receiving venetoclax and obinutuzumab in combination, safety and efficacy data including frequencies of AEs, response rates, and survival data were very similar to findings of our study and the MURANO trial [21]. They reported no cases of clinical TLS and only one (3%) patient experienced biochemical TLS. These RW data confirm the favorable risk profile and efficacy as observed in the CLL14 and MURANO trials indicating similar findings in RCTs and in RW cohorts with comparable patient characteristics and not heavily pretreated patients. A recent study aimed to evaluate the impact of age, comorbidity, and other patients' characteristics on outcomes in R/R CLL patients receiving venetoclax in a RW setting [22]. They found no associations with discontinuation or permanent dose reduction of venetoclax and only ECOG performance status had a negative influence on survival data. In a study of R/R CLL patients heavily pretreated with a median of 3 lines of prior therapies and the majority having adverse genetic features, dose escalation of venetoclax to 400 mg was achieved in 85% of patients [11]. TLS was reported in 13%, neutropenia in 47%, and ORR in 72%. In a French study reporting RW data on patients with similar unfavorable prognostic characteristics, a TLS rate of 22% and a 1-year PFS of 61% were observed [23]. These data suggest that dose intensity, toxicity, and response rates are to some extent dependent on the number of previous treatments, and this category of patients can be expected to be understated in RCTs.

Our data have limitations due to the retrospective nature with a limited number of patients. The cross-study comparisons are vulnerable to bias and confounding, but compared with RCTs, the core aspect of RW experience is its complementary nature rather than a tightly controlled comparison. In conclusion, our data demonstrated favorable and comparable safety and efficacy profiles of venetoclax in our single-institution RW and RCT patients. The data were also in line with data reported in the multicenter key RCTs of venetoclax in both treatment-naïve and R/R CLL patients. Furthermore, TLS was not a challenge in our RW patients with the current recommended precautions, and our TLS monitoring approach, omitting 6-h and 12-h TLS biochemistry, was safe and made the ramp-up procedure more feasible. Compared to patients in RCTs, our patients treated outside a clinical trial had more infections, and prophylactic antibiotics must be carefully considered when treating patients with venetoclax in the standard clinical practice.

## Figures and Tables

**Table 1:** Baseline demographics and clinical CLL characteristics according to whether the treatment was part of a clinical trial or real-life standard practice.

Baseline characteristics	Patients treated outside a clinical trial (*n* = 74)	Patients treated in a clinical trial (*n* = 38)	Total cohort (*n* = 112)
Demographics (*n*, %)			
Male	51 (69)	29 (76)	80 (71)
Female	23 (31)	9 (24)	32 (29)
Age (median, range)	74 (53–89)	70 (47–86)	72 (47–89)
CLL clinical characteristics (*n*, %)			
Treatment-naïve	23 (31)	17 (45)	40 (36)
B-symptoms	45 (61)	26 (68)	71 (63)
Lymphadenopathy	71 (96)	36 (95)	107 (96)
Splenomegaly	48 (65)	26 (68)	74 (66)
Hepatomegaly	4 (5)	2 (5)	6 (5)
Binet Stage A	8 (11)	5 (13)	13 (12)
Binet Stage B	26 (35)	18 (47)	44 (39)
Binet Stage C	40 (54)	15 (40)	55 (49)
Comorbidities (*n*, %)			
CIRS-score (median, range)	6 (0–13)	5 (0–12)	6 (0–13)
Diabetes	10 (14)	6 (16)	16 (14)
Cardiovascular disease	29 (39)	17 (45)	46 (41)
Pulmonary disease	26 (35)	5 (13)	31 (28)
Active smoking	14 (19)	4 (11)	18 (16)
Renal disease	12 (16)	4 (11)	16 (14)
BMI (median, range)	26 (16–41)	26 (19–47)	26 (16–47)
Blood analyses (median, range)			
Hemoglobin (g/dL)	12 (6–16)	12 (7–15)	12 (6–16)
Leukocytes (10^9^/L)	39 (3–525)	54 (6–197)	46 (3–524)
Neutrophils (10^9^/L)	3 (1–17)	3 (1–8)	3 (1–17)
Lymphocytes (10^9^/L)	19 (1–314)	36 (1–156)	27 (1–314)
Thrombocytes (10^9^/L)	139 (15–667)	159 (51–316)	144 (15–667)
Creatinine (μmol/L)	86 (47–350)	85 (52–190)	85 (47–350)
ALT (U/L)	22 (8–220)	22 (12–66)	22 (8–220)
Alkaline phosphatase (U/L)	84 (44–1000)	76 (31–161)	82 (31–1000)
CRP (mg/L)	2 (0–110)	2 (0–18)	2 (0–110)
Beta-2-microglobuline (mg/L)	4 (2–7)	4 (2–7)	4 (2–7)
IgG (g/L)	7 (1–29)	5 (1–11)	6 (1–29)
Dohner FISH categories (*n*, %):			
17p deletion (or *TP53* disruption)	21 (29)	5 (13)	26 (23)
11q-deletion	24 (32)	8 (21)	32 (29)
Trisomy 12	6 (8)	4 (11)	10 (9)
Normal	7 (9)	9 (24)	16 (14)
13q-deletion	16 (22)	12 (32)	28 (25)
IGHV mutational status (*n*, %):			
IGHV unmutated	44 (60)	23 (61)	67 (60)

**Table 2:** Characteristics of venetoclax treatment in patients treated according to the standard practice or part of a clinical trial.

Treatment characteristics	Patients treated outside a clinical trial (*n* = 74)	Patients treated in a clinical trial (*n* = 38)	Total cohort (*n* = 112)
Treatment naïve prior to venetoclax (*n*, %)	23 (31)	17 (45)	40 (36)
Relapsed/refractory patients (*n*, %)	51 (69)	21 (55)	72 (64)
Lines of treatment prior to venetoclax (median, range)	1 (0–4)	1 (0–6)	1 (0–6)
Venetoclax + rituximab (*n*, %)	58 (78)	23 (61)	81 (72)
Venetoclax + obinutuzumab (*n*, %)	8 (11)	9 (24)	17 (15)
Venetoclax + ibrutinib (*n*, %)	0	9 (24)	9 (8)
Venetoclax + acalabrutinib (*n*, %)	0	2 (5)	2 (2)
Venetoclax monotherapy (*n*, %)	10 (14)	0	10 (9)
Maximum dose (400 mg) achieved (*n*, %)	64 (87)	34 (90)	98 (88)
Treatment duration in months (median, range)	13 (1–36)	12 (10–36)	13 (1–36)
Reduction of steady-state dose (*n*, %)	22 (30)	10 (26)	32 (29)
Discontinuation not preplanned (*n*, %)	17 (23)	10 (26)	27 (24)
Supportive G-CSF (*n*, %)	27 (37)	19 (50)	46 (41)
Antibiotic prophylaxis (*n*, %)	15 (20)	16 (42)	31 (28)

**Table 3:** Grades 3-4 AEs in patients treated with venetoclax in clinical trials or in routine clinical practice.

Grades 3-4 AE	Patients treated outside clinical trial (*n* = 74)	Patients treated in clinical trial (*n* = 38)	Total cohort (*n* = 112)
*Infections*			
Pneumonia	19 (26)	3 (8)	22 (20)
Upper respiratory infection	1 (1)	1 (3)	2 (2)
Sepsis	3 (4)	1 (3)	4 (4)
Urinary tract infection	4 (5)	2 (5)	6 (5)
Febrile neutropenia	2 (3)	2 (5)	4 (4)

*Hematologic events*			
Neutropenia	31 (42)	20 (53)	51 (46)
Anemia	5 (7)	1 (3)	6 (5)
Thrombocytopenia	6 (8)	1 (3)	7 (6)
Bleeding	1 (1)	1 (3)	2 (2)

*Nonspecific symptoms*			
Diarrhea	2 (3)	0	2 (2)
Nausea	1 (1)	0	1 (1)
Fatigue	2 (3)	1 (3)	3 (3)
Vertigo	0	1 (3)	1 (1)
Hypotension	1 (1)	0	1 (1)
Arthralgia	1 (1)	1 (3)	2 (2)

*Note:* Values are numbers and percentages.

## Data Availability

For original data, please contact the corresponding author. As the informed consent does not allow disclosure of individual patients' data, only aggregated data can be provided upon request. The datasets are not publicly available due to the National and European Data Protection Regulation.
